# Insulin enhances striatal dopamine release by activating cholinergic interneurons and thereby signals reward

**DOI:** 10.1038/ncomms9543

**Published:** 2015-10-27

**Authors:** Melissa A. Stouffer, Catherine A. Woods, Jyoti C. Patel, Christian R. Lee, Paul Witkovsky, Li Bao, Robert P. Machold, Kymry T. Jones, Soledad Cabeza de Vaca, Maarten E. A. Reith, Kenneth D. Carr, Margaret E. Rice

**Affiliations:** 1 Department of Neuroscience and Physiology, New York University School of Medicine, 550 First Avenue, New York, New York 10016, USA; 2 Department of Neurosurgery, New York University School of Medicine, 550 First Avenue, New York, New York 10016, USA; 3 Center for Neural Science, New York University, 4 Washington Place, New York, New York 10003, USA; 4 Department of Ophthalmology, New York University School of Medicine, 550 First Avenue, New York, New York 10016, USA; 5 Smilow Neuroscience Program, New York University School of Medicine, 550 First Avenue, New York, New York 10016, USA; 6 Department of Psychiatry, New York University School of Medicine, 550 First Avenue, New York, New York 10016, USA; 7 Department of Biochemistry and Molecular Pharmacology, New York University School of Medicine, 550 First Avenue, New York, New York 10016, USA

## Abstract

Insulin activates insulin receptors (InsRs) in the hypothalamus to signal satiety after a meal. However, the rising incidence of obesity, which results in chronically elevated insulin levels, implies that insulin may also act in brain centres that regulate motivation and reward. We report here that insulin can amplify action potential-dependent dopamine (DA) release in the nucleus accumbens (NAc) and caudate–putamen through an indirect mechanism that involves striatal cholinergic interneurons that express InsRs. Furthermore, two different chronic diet manipulations in rats, food restriction (FR) and an obesogenic (OB) diet, oppositely alter the sensitivity of striatal DA release to insulin, with enhanced responsiveness in FR, but loss of responsiveness in OB. Behavioural studies show that intact insulin levels in the NAc shell are necessary for acquisition of preference for the flavour of a paired glucose solution. Together, these data imply that striatal insulin signalling enhances DA release to influence food choices.

## Abstract (web summary)

Insulin signals satiety after a meal; however, the rising incidence of obesity and chronic insulin elevation suggests that insulin may also signal reward. Here, Stouffer *et al*. show that insulin amplifies dopamine release in rodent striatum depending on diet, and that striatal insulin can influence food choice.

## Graphical abstract

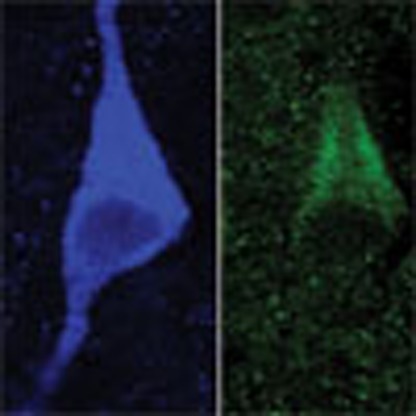

It is well established that a sustained increase in plasma insulin during and after a meal activates insulin receptors (InsRs) in the hypothalamus, which provides negative feedback to appetitive circuits that decreases further eating^1^,^2^,^3^. Brain insulin is derived primarily from pancreatic β cells, with active transport from plasma to brain at the blood–brain barrier^4^,^5^,^6^,^7^,^8^, although there is increasing evidence for neuronal insulin synthesis and release, as well^1^,^9^. Notably, expression of InsRs is not confined to the hypothalamus, although the function of extra-hypothalamic InsRs remains unresolved^1^,^2^,^3^. Given the rising incidence of obesity and type II diabetes, in which circulating levels of insulin are perpetually elevated and brain insulin transport and receptor sensitivity are decreased^3^,^8^,^10^,^11^, it is critical to understand the function of insulin in brain regions that regulate motivation and reward. Brain regions of particular interest include the nucleus accumbens (NAc), which mediates the rewarding effects of both food and drugs^12^,^13^, and the caudate–putamen (CPu), which plays a role in habit-based behaviours and craving^13^. InsRs are expressed throughout these regions, with the highest density occurring in the NAc^3^,^14^; InsRs are also expressed by dopamine (DA) neurons in the midbrain, including those in the ventral tegmental area (VTA) and substantia nigra pars compacta (SNc)^15^. Brain insulin levels are proportional to plasma insulin concentrations and to body adiposity^6^,^7^,^8^, leading to the hypothesis that insulin might act at InsRs in these brain regions to influence food reward^3^,^16^,^17^.

Previous studies in striatal synaptosomes, heterologous cells, brain slices and *in vivo* have shown that insulin activation of InsRs leads to an increase in DA uptake by the DA transporter (DAT)^18^,^19^,^20^,^21^,^22^,^23^. This process involves the PI3 kinase signalling pathway^19^,^20^, and results in DAT insertion in the plasma membrane^19^. Circulating insulin levels dynamically modulate striatal DAT activity, with decreased DA uptake and DAT surface expression seen in animal models of diabetes and after food restriction (FR)^20^,^21^. Insulin-dependent increases in DAT activity have been shown to decrease evoked extracellular DA concentration ([DA]_o_) in the VTA^23^, reflecting a shift in the balance between DA release and uptake. Consistent with the established role of insulin in satiety, acute microinjection of insulin in the VTA can decrease food reward^23^,^24^, whereas mice lacking InsRs in VTA and SNc DA neurons show increased food intake and become obese^25^. Although insulin can induce long-term depression of excitatory input to VTA DA neurons^24^, again consistent with a role in satiety, insulin exposure can also increase DA neuron firing rate, possibly by decreasing DA release and autoreceptor-mediated inhibition^25^. The net effect of insulin on striatal DA release is therefore hard to predict. Indeed, the results from studies of the influence of insulin on striatal DA release in *ex vivo* slices^19^ and the effect of local microinjection of insulin in the NAc on food reward^26^ appear to be contradictory. To resolve this, we evaluated axonal DA release and uptake in the intact microenvironment of the NAc and CPu in *ex vivo* striatal slices using fast-scan cyclic voltammetry (FCV), and determined the effects of insulin signalling in the NAc on reward behaviour *in vivo*.

Our studies show that the primary effect of insulin in NAc and CPu is to enhance DA release, despite a concurrent increase in DA uptake. This dynamic regulation of DA release involves an insulin-dependent increase in the excitability of striatal cholinergic interneurons (ChIs), which leads to enhanced DA release via activation of nicotinic acetylcholine (ACh) receptors (nAChRs). The influence of insulin on ChIs and on DA release is mediated by InsRs. Notably, the effect of insulin on DA release is amplified in slices from FR rats, but blunted in rats on an obesogenic (OB) diet. These data showing amplification of DA release in *ex vivo* slices by insulin lead to the prediction that insulin might act as a reward signal *in vivo*. Indeed, parallel behavioural studies demonstrate a role for insulin in the NAc shell in flavour-preference conditioning. Together, these findings indicate a new role for insulin as a reward signal that can influence food choice.

## Results

### Insulin acting at InsRs increases evoked striatal DA release

**Figure 1:**
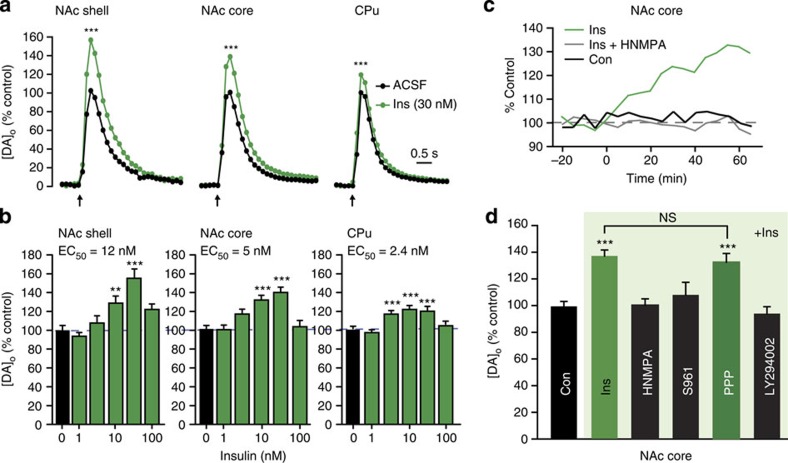
Insulin-dependent increases in evoked [DA]_o_ require InsRs and PI3K. (**a**) Average single-pulse-evoked [DA]_o_ in NAc shell, NAc core and CPu before and after insulin (Ins) illustrated for 30 nM; error bars omitted, but see (**b**); arrows indicate time of stimulus. Insulin increased evoked [DA]_o_ in shell (by 55±10%), core (by 37±5%) and CPu (by 20±4%) (***P<0.001). (**b**) Effect of insulin was concentration dependent across a physiological range (1–30 nM) in shell (*n*=22–24, *F*_5,133_=14.471, *P*<0.001), core (*n*=36–76, *F*_5,308_=16.318, *P*<0.001) and CPu (*n*=30–62, *F*_5,253_=13.763, *P*<0.001), but lost at ≥100 nM. (**c**) Representative recordings of peak-evoked [DA]_o_ versus time at a single site in the NAc core in the absence of drug application (Con), during application of insulin (30 nM) or when insulin was applied in the presence of an InsR inhibitor HNMPA (5 μM). (**d**) Average peak-evoked [DA]_o_ data showing prevention of the effect of insulin (30 nM) by HNMPA, InsR antagonist S961 (1 μM) and PI3K inhibitor LY294002 (1 μM), but not by IGF-1R inhibitor PPP (1 μM) (*n*=29–76; *P*>0.9 versus insulin alone). For Fig. 1a–d, *n*=number of sites per subregion from 3–6 rats for each drug or insulin concentration; one-way ANOVA, Tukey honest significance test (HSD). See Supplementary Fig. 1b,c for NAc shell and CPu data.

Initial examination of locally evoked [DA]_o_ monitored with FCV in *ex vivo* striatal slices from rats with *ad libitum* (AL) access to food and water revealed the unexpected finding that acute application of insulin across a range of physiologically relevant concentrations^1^,^4^ increased single-pulse-evoked [DA]_o_ (Fig. 1a–c), with insulin EC_50_ values (the concentration at which the effect was half maximal) of 2–12 nM (Fig. 1b). Increased evoked [DA]_o_ was particularly surprising, given that this was accompanied by a significant increase in the maximal rate (*V*_max_) for DAT-mediated uptake in each subregion (Table 1), which would lead to a competing decrease in evoked [DA]_o_, as reported previously^22^,^23^. Instead, we found that evoked [DA]_o_ was maximally amplified 20–55% by 30 nM insulin; the region with the greatest proportional effect was the NAc shell, which is the striatal subregion with highest InsR expression^1^,^14^. Slices exposed to 30 nM insulin under identical conditions showed no change in striatal DA content (Supplementary Fig. 1a), implying that insulin alters dynamic release regulation, rather than simply upregulating DA synthesis. Notably, the effect of insulin on evoked [DA]_o_ was lost at supraphysiological concentrations ≥100 nM (Fig. 1b). This was not a result of increased DAT activity overtaking release, as the effect of insulin on *V*_max_ was also lost at these concentrations (Table 1). Overall, these data show that in the intact striatal microenvironment, the predominant effect of insulin on evoked [DA]_o_ is to increase release, despite a concurrent increase in DA uptake.

Because insulin can also act at insulin-like growth factor 1 receptors (IGF-1Rs), albeit at concentrations exceeding 100 nM (ref. 1), we sought to confirm that the enhancing effect of insulin on evoked [DA]_o_ was InsR dependent. This proved to be the case, as the effect was prevented by an intracellular InsR inhibitor, hydroxy-2-naphthalenylmethylphosphonic acid (HNMPA), and by an InsR antagonist, S961, but not by a selective inhibitor of IGF-1Rs, picropodophyllin^24^ (PPP; Fig. 1c,d and Supplementary Fig. 1b,c). We then examined the involvement of PI3 kinase, which initiates the signalling pathway responsible for insulin-dependent regulation of the DAT^19^. At a concentration of 1 μM, the P13K inhibitor LY249002 alone had no effect on peak-evoked [DA]_o_ or *V*_max_ (*n*=29–76 sites (NAc core) per drug, *P*>0.05, one-way analysis of variance (ANOVA); data not shown), yet prevented the effect of insulin on evoked [DA]_o_ in all striatal subregions (Fig. 1d and Supplementary Fig. 1b,c).

### Localization of InsRs on DA axons and ChIs

**Figure 2:**
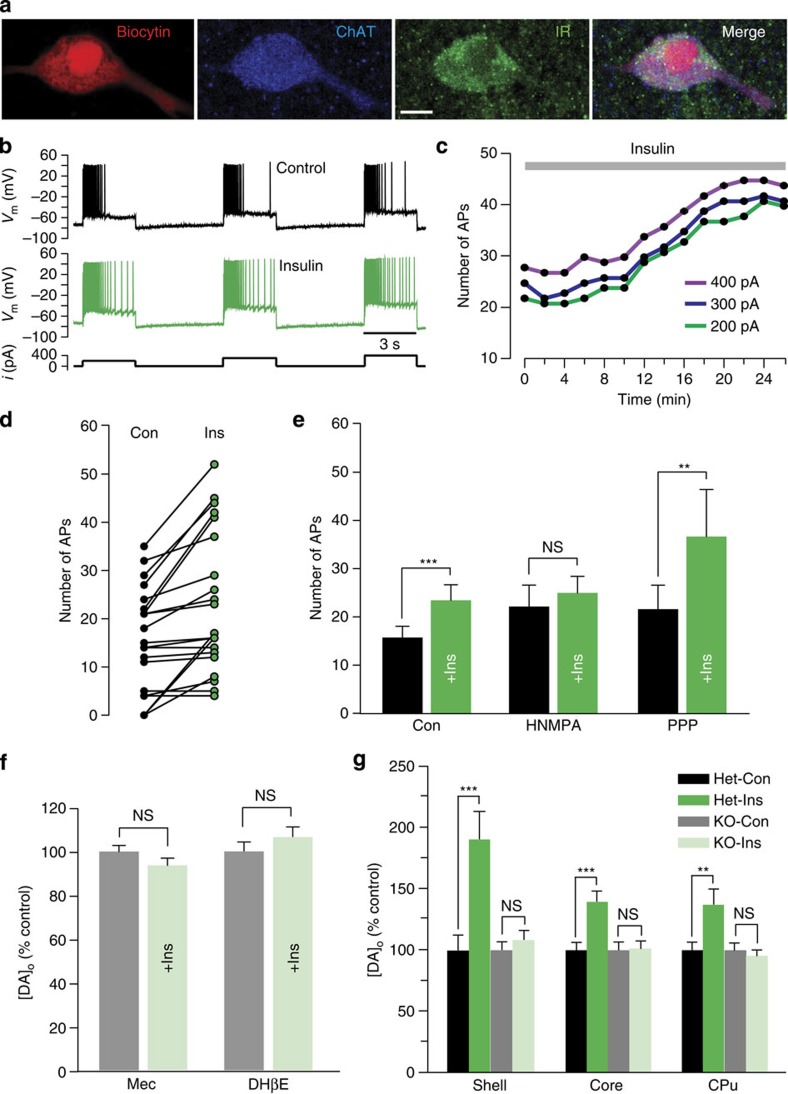
Insulin-dependent regulation of striatal DA release requires ACh from ChIs. (**a**) ChI filled with biocytin, then immunolabelled for ChAT, and InsR (representative of 4/4 biocytin-filled ChIs); merged image shows co-localization; scale bar, 10 μm. (**b**–**e**) Response of striatal ChIs to a series of depolarizing current pulses (3-s duration; 200, 300 and 400 pA; 120-s intervals) before and after insulin (30 nM). (**b**) Spike frequency adaptation in a ChI (upper) is seen in loss of action potential (AP) discharge during current injection, whereas spiking persists throughout the current pulse in insulin (lower); complete data set shown in **d**. (**c**) Representative time course of the insulin-induced increase in AP number with each current step for the ChI in **b**. (**d**) Summary of AP number during current pulses delivered before and at the peak effect of insulin exposure (*n*=21 paired stimulations, 7 neurons, 5 rats) (**e**) Mean responses showing the effect of insulin (+Ins) under control conditions (Con; *n*=21 paired stimulations, 7 neurons, ***P<0.001, paired two-tailed *t*-test), in the presence of HNMPA (5 μM) (*n*=12 paired stimulations, 4 neurons, 4 rats, *P>*0.05, paired two-tailed *t*-test), and in the presence of PPP (1 μM) (*n*=18 paired stimulations, 6 neurons, 6 rats, **P<0.01, Wilcoxon matched pair signed rank test). (**f**) Average single-pulse-evoked [DA]_o_ in NAc core before and after insulin (30 nM) in mecamylamine (Mec; 5 μM) or DHβE (1 μM) normalized to 100% peak control (*n*=20–40 sites per subregion per condition from 3–4 rats, *P*>0.05 versus control, unpaired *t*-test). (**g**) Average single-pulse-evoked [DA]_o_ in forebrain slices from heterozygous control (Het) and *ChAT* KO mice before and after insulin (30 nM), normalized to 100% peak control. Insulin increased evoked [DA]_o_ in heterozygous mice by 190±23% in NAc shell, 140±8% in NAc core and 137±12% in CPu (*n*=15–25 sites per subregion from 3–4 mice per genotype, **P<0.01, ***P<0.001 versus control unpaired *t*-test), but had no effect on evoked [DA]_o_ in any striatal subregion of *ChAT* KO mice (*P*>0.1).

The observed increase in *V*_max_ for DA uptake with physiological levels of insulin implies the presence of InsRs on DA axons, as do previous results in various striatal preparations^18^,^19^,^20^,^21^,^22^. Although functional expression of InsRs on midbrain DA neurons has been demonstrated^15^,^23^,^24^,^25^, InsR expression on striatal DA axons has not been reported. We addressed this using immunohistochemistry. Dense InsR immunoreactivity throughout the striatum limited quantitative assessment of InsR localization on DA axons, which were identified by immunoreactivity for a DA-synthesizing enzyme, tyrosine hydroxylase (TH). We therefore adopted a previously reported protocol^27^, which involved counting InsR puncta that overlapped with TH+ profiles in normal image view and counting again after the InsR image only was rotated by 90°. If apparent overlap of InsR and TH+ profiles were nonspecific, this procedure should give statistically similar counts whether normal or 90° out of phase. However, this analysis showed a decrease in the overlap of InsR puncta with TH+ profiles of 14±9% (*n*=42 fields, *P*<0.01, paired two-tailed *t*-test; data not shown), confirming InsR presence on DA axons. More intriguingly, however, InsR immunolabelling of the striatum revealed distinct InsR expression on large cell bodies that were identified as striatal ChIs by co-immunolabelling for choline acetyltransferase (ChAT), the primary enzyme required for ACh synthesis. Using electrophysiological criteria^28^ to identify ChIs in preliminary whole-cell-recording studies, several neurons were filled with biocytin and then processed for immunohistochemistry; all of these (4/4) were immunopositive for both InsR and ChAT (Fig. 2a). Subsequent evaluation of InsR and ChAT co-localization in the NAc confirmed that virtually all ChAT+ neurons expressed InsR (96%; *n*=27/28 neurons in four sections from two rats).

### Insulin increases ChI excitability

To test the functionality of InsRs on striatal ChIs, we examined the effect of insulin on ChI excitability using whole-cell current-clamp recording. ChI excitability was assessed using a series of 3-s depolarizing current pulses to elicit a train of action potentials that reliably exhibited spike frequency adaptation (Fig. 2b), often with loss of spiking by the end of the current pulse. Strikingly, insulin (30 nM) attenuated spike frequency adaptation, resulting in a progressive increase in action potential number over time (Fig. 2c), with a maximum increase (Fig. 2d,e) typically seen between 20 and 50 min of insulin exposure. In the absence of insulin, control ChIs showed no change in the number of evoked action potentials (*P*>0.05, paired two-tailed *t*-test; data not shown); when compared with control neurons monitored over the same time interval, neurons exposed to insulin showed a significantly greater change in the number of evoked action potentials (control *n*=12 stimulus pairs from four neurons, insulin *n*=21 stimulus pairs from seven neurons, *F*_1,25_=5.63, *P*<0.05, mixed-measures two-way ANOVA; data not shown). The effect of insulin on increasing action potential number was prevented by HNMPA but not by the IGF-1R selective inhibitor PPP (Fig. 2e), demonstrating that increased ChI excitability by insulin was InsR mediated.

### Insulin enhancement of evoked [DA]_o_ requires nAChRs and ACh

Previous studies have shown that ChIs and ACh potently regulate striatal DA release via nAChRs on DA axons^29^,^30^,^31^,^32^,^33^,^34^. Abundant InsR expression on ChIs and the enhancement in ChI excitability seen with acute insulin exposure suggested that these neurons might be novel targets for insulin that could lead to enhanced DA release. To test this, we examined the effect of insulin in the presence of mecamylamine, a non-selective nAChR antagonist, or dihydro-β-erythroidine (DHβE), a selective antagonist for β2 subunit-containing (β2*) nAChRs that are enriched on DA axons^35^. Evoked [DA]_o_ was readily detected in the presence of these antagonists, even though both drugs decreased the amplitude of single-pulse-evoked [DA]_o_ (for example, by 13–26% in NAc core), as reported previously^29^,^30^,^31^,^32^. In support of a role for ACh and nAChRs, the effect of insulin on evoked [DA]_o_ was prevented by either mecamylamine or DHβE (Fig. 2f). To confirm the involvement of striatal ACh signalling in insulin-enhanced DA release, we examined the effect of insulin in *ex vivo* striatal slices from mice in which ChAT expression was genetically ablated in forebrain structures (forebrain *ChAT* KO mice), including striatum^32^. Although ChIs are intact in these mice, ACh synthesis is abolished, which leads to decreased, but still readily detectable single-pulse-evoked [DA]_o_, as described previously^32^. In control heterozygous littermates, insulin (30 nM) increased evoked [DA]_o_ in NAc shell and core and in CPu by 37–90% (Fig. 2g), exceeding the amplification seen in rat striatum (for example, Fig. 1). However, the effect of insulin on evoked [DA]_o_ was absent throughout the striatal complex in forebrain *ChAT* KO mice, demonstrating that insulin-mediated enhancement of DA release requires striatal ACh, but not co-released transmitters from ChIs, such as glutamate^36^.

### The influence of insulin on evoked [DA]_o_ is diet dependent

**Figure 3:**
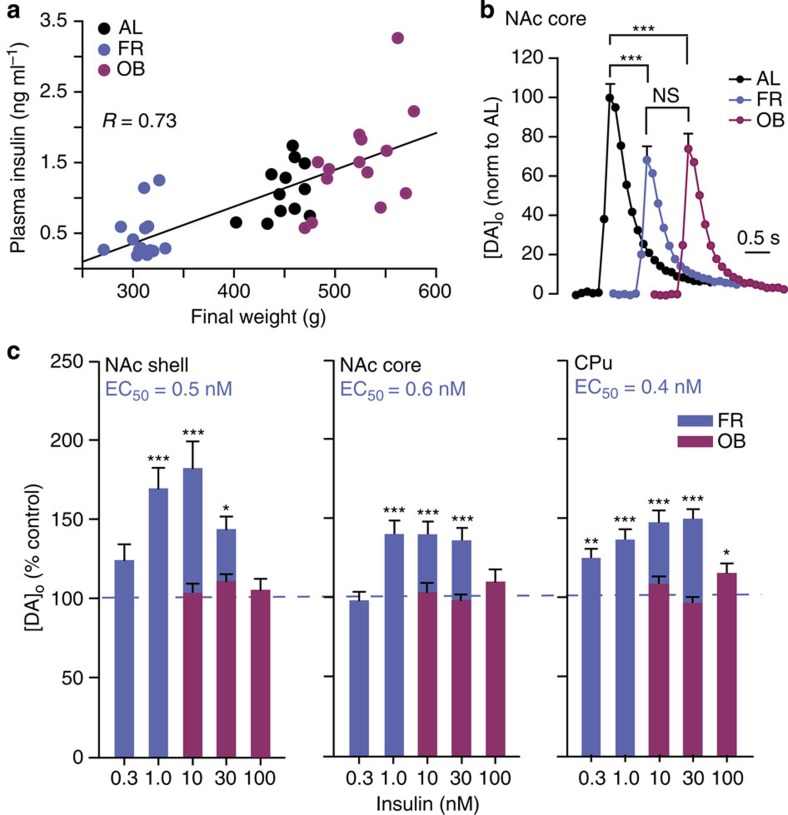
Insulin-induced increases in evoked [DA]_o_ are enhanced by FR and lost in OB. (**a**) Plasma insulin concentration is positively correlated with body weight across feeding groups (*R*=0.76). (**b**) Average single-pulse-evoked [DA]_o_ in NAc core (see Supplementary Fig. 2a,b for NAc shell and CPu) was lower in FR (38±4%) and OB (25±4%) versus AL (*n*=50–60 sites from 5–6 rats per diet group, *F*_2,156_=23.337, one-way ANOVA, Tukey HSD; ***P<0.001); OB versus FR (*P<*0.08). (**c**) Sensitivity of evoked [DA]_o_ to insulin was enhanced in FR, but lost in OB (*n*=21–49 sites per subregion per concentration from 2–4 rats per diet group, one-way ANOVA, Tukey HSD), with greater sensitivity in FR versus AL rats in all subregions (*P*<0.001 for each region; two-way ANOVA; CPu: *F*_(conc × diet; 3,286)_=10.253; core: *F*_(conc × diet; 3,353)_=6.166; shell: *F*_(conc × diet; 3,195)_=10.735).

Plasma and brain insulin concentrations are proportional to body adiposity^6^,^7^,^8^, and could lead to compensatory changes in brain sensitivity to insulin. We therefore tested the hypothesis that diet influences the ability of insulin to promote DA release, using striatal slices from rats maintained on either a chronic FR or OB diet versus AL controls. As expected, plasma insulin levels were correlated with body weight, with lower insulin in FR than in AL or OB rats (Fig. 3a and Table 2). Despite these differences in circulating insulin, peak-evoked [DA]_o_ in NAc shell and core, and CPu was significantly lower in *ex vivo* striatal slices from both diet groups compared with AL (Fig. 3b and Supplementary Fig. 2 a,b), implying that factors in addition to insulin govern absolute evoked [DA]_o_. Striatal DA content did not differ among diet groups, indicating a change in release regulation rather than in DA synthesis (Supplementary Fig. 2c). Consistent with a change in dynamic regulation, the sensitivity of striatal DA release to insulin was markedly diet dependent. In FR rats, insulin concentrations ≤1 nM, which had no effect in AL (Fig. 1b), increased evoked [DA]_o_ (Fig. 3c), reflecting increased insulin sensitivity, with EC_50_ values in FR striatum (0.4–0.6 nM) that were approximately an order of magnitude lower that in AL (compare Figs 1b and 3c). In striking contrast, the effect of insulin was lost in OB striatum; even 30 nM insulin, which had a maximal effect in AL striatum (Fig. 1b), had no effect in OB (Fig. 3c).

These data imply an inverse relationship between striatal InsR sensitivity and body adiposity. Alternatively, however, these diet-dependent differences might reflect altered nAChR sensitivity. We therefore determined the concentration response to nicotine in NAc core from each diet group. Nicotine causes nAChR desensitization, which can be quantified by comparing the ratio of [DA]_o_ evoked by a brief train of five pulses at 100 Hz to single-pulse-evoked [DA]_o_ (5 p:1 p ratio) as an index of nAChR activation/desensitization^30^,^31^. Using this approach, we found no differences among diet groups in nAChR sensitivity in the NAc core (Supplementary Fig. 3a–c). Furthermore, control 5 p:1 p ratio did not differ among diet groups in NAc core (Supplementary Fig. 3d) or CPu (not illustrated), implying that diet does not alter nAChR-dependent DA release regulation. Thus, striatal InsR sensitivity appears to be enhanced in FR versus AL rats, but absent in OB rats, with loss of regulation of striatal DA release at physiological concentrations of insulin.

### NAc shell insulin modulates conditioned flavour preference

Food preferences are generated by both pre- and post-ingestive factors; the mechanisms for each are not completely resolved, but current evidence implicates NAc DA signalling in both^37^,^38^. Given that plasma and cerebrospinal fluid (CSF) insulin levels rise rapidly after peripheral glucose elevation^6^, and that an increase in insulin in the striatum can be detected within 5 min of plasma insulin elevation^7^, it is logical to hypothesize that peripheral insulin release during a meal could enhance NAc DA release and contribute to post-ingestive reward mechanisms. We adapted a previously described flavour-preference protocol^37^ with saccharin-sweetened glucose solutions in rats to test the hypothesis that blocking the effect of endogenous insulin through local application of an insulin antibody (InsAb) in the NAc would decrease preference for the paired flavour. The efficacy of the InsAb in blocking the effects of insulin was tested in an *in vitro* assay of DA uptake into striatal synaptosomes. Insulin (30 nM) caused a significant increase in *V*_max_ in synaptosomes from NAc or CPu (Supplementary Fig. 4), consistent with our *V*_max_ data from striatal slices (Table 1) and with previous studies^18^,^19^,^20^,^21^,^22^,^23^. In the absence of insulin, neither InsAb nor a control antibody immunoglobulin G (IgG) altered the *V*_max_ for DA uptake versus control. In the presence of IgG, insulin still caused a significant increase in *V*_max_; however, the effect of insulin on *V*_max_ was lost in the presence of InsAb (Supplementary Fig. 4).

**Figure 4:**
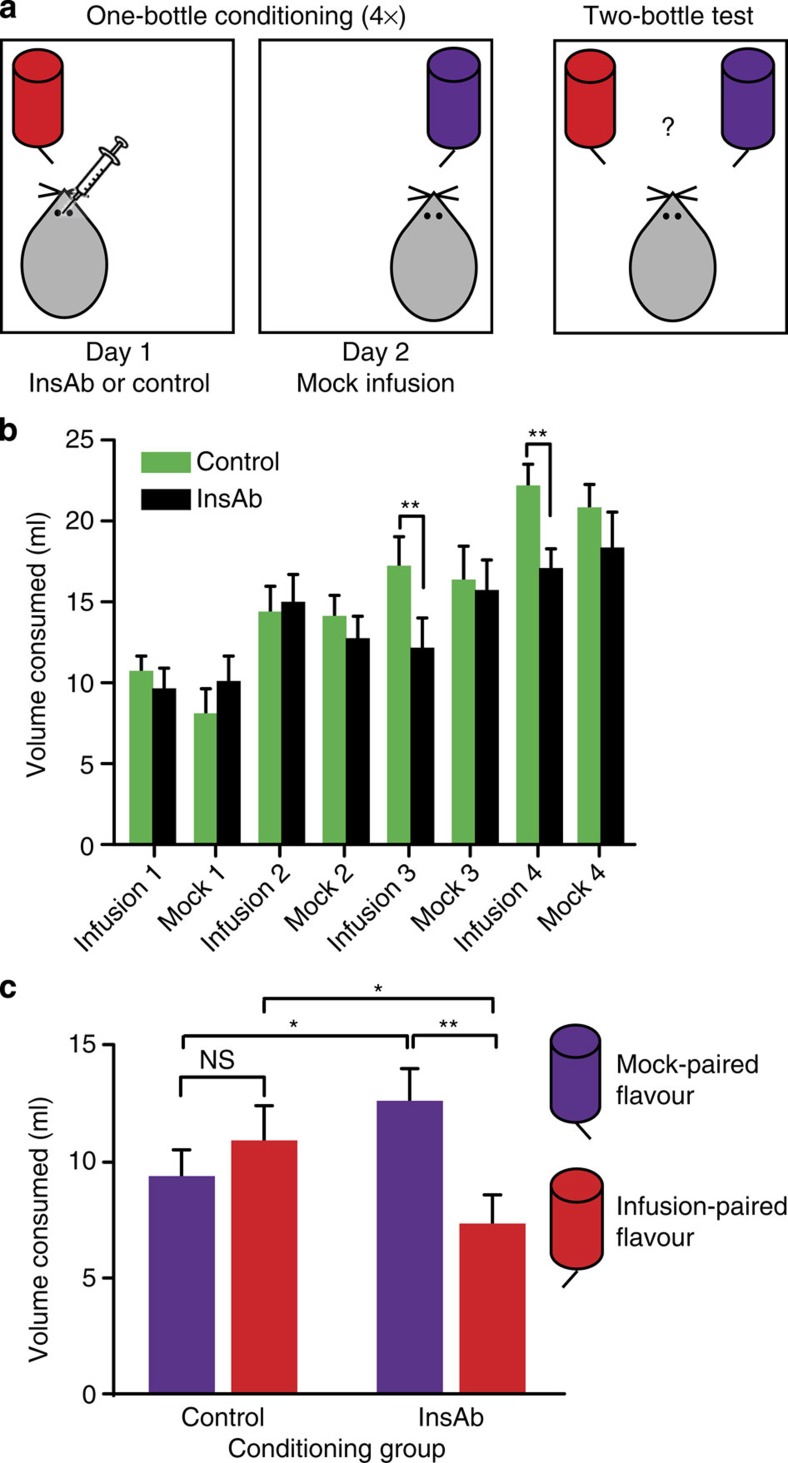
InsAb microinjection into the NAc shell decreases flavour preference. (**a**) Diagram illustrating one-bottle conditioning (left) and the two-bottle test (right). (**b**) Volume consumed (ml) during one-bottle-conditioning sessions. There was a significant interaction between infusion-conditioning session and microinjection treatment (*n*=19–20 rats per group, *F*_(3,111)_=3.088, *P*<0.05, 2 × 4 mixed ANOVA with repeated measures on infusion-conditioning session). InsAb microinjection significantly decreased consumption compared with control during the third (*t*(40)=3.026, **P<0.01) and fourth (*t*(40)=3.052, **P<0.01, protected one-tailed *t*-tests) infusions. Mock injections had no effect on consumption in either group (*F*_3,111_=1.110, 2 × 4 mixed ANOVA with repeated measures on mock conditioning session). (**c**) Volume consumed during two-bottle flavour-preference test. There was a significant interaction between flavour and microinjection treatment during conditioning (*F*_1,37_=5.36, *P*<0.05, two-way mixed ANOVA with repeated measures on flavour). The InsAb group consumed significantly less of the InsAb-paired flavour compared with the mock-paired flavour (*t*(18)=2.82, **P<0.01, protected one-tailed *t*-test); the control group showed no flavour preference (*t*(19)=0.803, *P*>0.05, protected *t*-test). Comparing groups, InsAb rats drank significantly less of the infusion-paired flavour (*t*(40)=1.96, *P<0.05) and significantly more of the mock-paired flavour (*t*(40)=1.77, *P<0.05, protected one-tailed *t*-test) than controls.

To minimize tissue damage and preserve sensitivity of the tissue target, two groups of subjects were tested in which we alternated intra-NAc microinjection with a mock microinjection procedure, rather than using a single group of subjects and pairing one flavoured solution with InsAb and another with vehicle. Consequently, during one-bottle-conditioning sessions, the experimental group received InsAb microinjections paired with one of two flavours, and on alternating sessions, mock microinjections paired with the other flavour (Fig. 4a, left). The control group received mock microinjections alternated with microinjections of either phosphate-buffered saline (PBS) or IgG. Both flavoured solutions contained glucose during conditioning. No differential preference between flavours was expected in the control-microinjected group, whereas preference was expected to shift toward the mock microinjection-paired flavour in the InsAb-microinjected group.

During the one-bottle conditioning sessions, InsAb microinjection significantly decreased consumption compared with vehicle during the third and fourth infusions (Fig. 4b). By contrast, both groups consumed the same volume of solution during all four mock injection sessions (*F*_3,111_=0.127, *P>*0.05, mixed two-way ANOVA) (Fig. 4b). After a total of eight conditioning sessions, flavour preference was assessed in a two-bottle test in which rats had access to both flavoured solutions simultaneously (Fig. 4a). Statistical analysis revealed a significant interaction between flavour and the microinjection treatment received during conditioning (Fig. 4c). The InsAb group consumed significantly less of the InsAb-paired flavour compared with the mock-paired flavour (Fig. 4c), whereas the vehicle group showed no flavour preference (Fig. 4c), implying that intact insulin signalling contributed to selection of a sweet caloric solution. Compared with vehicle, InsAb-microinjected rats drank significantly less of the infusion-paired flavour and significantly more of the mock-injection-paired flavour (Fig. 4c). Microinjection of IgG (*t*(9)=0.792. *P>*0.05, protected *t*-tests) or PBS (*t*(9)=0.442. *P>*0.05, protected *t*-tests) had no effect on flavour preference (data not shown), arguing against the possibility that a nonspecific effect of InsAb microinjection decreased consumption or flavour preference. It should also be noted that the preference of the InsAb group at test is not a preference for the less novel flavour, as there was no interaction between session and session type (actual infusion versus mock infusion) for the InsAb group (*F*_3,54_=1.584, *P*>0.05, two-way ANOVA). That is, the InsAb group did not consume more of the mock-infusion-paired flavour relative to the InsAb infusion-paired flavour; rather, differences between treatment groups only emerged during infusion-conditioning sessions. Overall, these data indicate that insulin in the NAc plays a role in reinforcement of preference for a flavour that signals a glycaemic load.

## Discussion

We report here that insulin amplifies striatal DA release in a nAChR-dependent manner by modulating ChI excitability via InsRs. Our results imply that insulin can serve as a reward signal, in addition to its established role in signalling satiety. Notably, the effect of insulin on DA release is modulated by diet, with markedly increased sensitivity to insulin after FR, but a complete loss of insulin-enhanced regulation on an OB diet. These changes appear to reflect changes in InsR sensitivity that are inversely related to circulating insulin levels, given that plasma insulin levels were found to be diet dependent, but nAChR sensitivity was not. Finally, our flavour-preference studies in behaving animals imply that insulin signalling in the NAc shell influences food preference, which not only implicates insulin in food-related learning but also confirms its role as a reward signal.

Net [DA]_o_ reflects the balance between DA release and DA uptake via the DAT. Previous evidence showing that insulin can regulate DAT activity^18^,^19^,^20^,^21^,^22^,^23^ led to the prediction that an increase in insulin should cause a net decrease in evoked [DA]_o_ via increased DA uptake. However, we found that in the striatum, the effect of insulin is more complex than this. Although insulin exposure did increase *V*_max_ for the DAT, the primary effect of insulin was on DA release, not on DA uptake, with a consistent increase in evoked [DA]_o_ across a physiological range of insulin concentrations in NAc shell and core and in CPu. Although the increase in evoked [DA]_o_ did revert to control levels at a supraphysiological concentration of 100 nM, *V*_max_ was also unchanged from control, eliminating a predominant effect on the DAT as an explanation. Instead, loss of the effect on uptake as well as release implicates desensitization of InsRs or downregulation of downstream signalling pathways at high insulin concentrations. Indeed, InsRs undergo rapid endocytosis and degradation after insulin binding in peripheral tissues^1^, with emerging evidence for loss of neuronal InsR sensitivity after short-term exposure to high levels of insulin or high-calorie diets^10^,^11^,^39^.

The dominant effect of insulin to enhance striatal DA release reported here contrasts with the results of two other recent *ex vivo* slice studies. In the first, insulin caused a decrease in electrically evoked overflow of [^3^H]DA from striatal slices, although increased [^3^H]DA overflow was detected when the DAT was inhibited^22^. Given that released [^3^H]DA must escape DAT-mediated uptake throughout the tissue to be detected in the superfusing solution, this protocol is especially sensitive to DAT regulation. Our results showing that insulin enhances DA release via ChIs and nAChR activation, in addition to enhancing DAT-mediated uptake, would explain the seemingly paradoxical increase in insulin-enhanced [^3^H]DA overflow seen when competing effects on the DAT were blocked^22^. The second study used FCV for direct detection of somatodendritic DA release in the VTA, but also found a predominant effect of insulin on DA uptake, reflected in decreased evoked [DA]_o_ (ref. 23). Differences in a number of factors, from local microcircuitry to somatodendritic versus axonal DA release mechanisms^40^, could contribute to this regional difference. As discussed further below, however, regionally dependent roles of insulin are likely to be complementary, rather than contradictory.

Previously, any role of insulin-dependent regulation of DA signalling had been assumed to be mediated by direct activation of InsRs on DA neurons. We show here that InsRs are also expressed on striatal ChIs, and that insulin modulates ChI excitability to amplify striatal DA release, which may play a pivotal role in the influence of insulin on diet. Striatal ChIs receive projections from neurons of the intralaminar nuclei of the thalamus, which exhibit burst firing in response to salient sensory stimuli and help drive burst-pause spiking patterns in ChIs that are important in directing attention, reinforcement and associative learning^41^. Therefore, the action of insulin at InsRs on striatal ChIs could enhance the effect of sensory food cues on striatal responsiveness to thalamic firing, contributing to increased perception of the reward value of an ingested meal. Direct promotion of striatal DA release by ChI activation has led to the suggestion that factors that stimulate ChIs will have a privileged role as triggers of DA release^33^. Our data provide the first supporting evidence for this, with insulin-enhanced ChI excitability and ACh signalling driving dynamic increases in DA release.

Elevated DA release in the presence of insulin also argues against an enhancement in ACh signalling to an extent that causes nAChR desensitization or muscarinic ACh receptor (mAChR) activation, either of which can suppress single-pulse-evoked [DA]_o_ (refs 29, 30, 31, 42). Thus, the mechanisms reported here are distinct from ACh activation of mAChRs, which has been associated with aversion and satiety^43^.

Single-pulse-evoked [DA]_o_ was lower in both FR and OB rats than in AL rats; although these results are in consonance with previous reports^44^,^45^,^46^, our studies provide the first systematic comparison of three striatal subregions in the two diet groups over a constant time frame. The mechanisms underlying diet-dependent changes in DA release have not been elucidated, and are beyond the scope of the present studies. However, given that plasma insulin levels are oppositely altered by FR and OB diets, it is unlikely that decreased evoked [DA]_o_ in both groups is a consequence of diet-dependent insulin levels.

On the other hand, changes in InsR sensitivity with diet and consequent diet-dependent plasma insulin levels provide the most likely explanation for increased sensitivity to insulin in FR and loss of responsiveness to insulin in OB. There was no evidence for the alternative explanation of altered nAChR sensitivity in either FR or OB rats versus AL. Although our findings are among the first to indicate increased striatal InsR sensitivity with FR^18^, weight loss can be accompanied by decreased insulin levels in CSF^7^, which would contribute to enhanced sensitivity of DA release to insulin in FR. Conversely, loss of insulin responsiveness in OB rats is consistent with previous evidence for decreased brain InsR sensitivity induced by weight gain or OB diets^3^,^10^,^11^.

Our *ex vivo* slice data support the hypothesis that insulin may signal reward as well as satiety. We tested this hypothesis by blocking the effect of endogenous insulin with bilateral InsAb microinjection in the NAc shell during flavour-preference conditioning. Consistent with a role in reward, blocking the effect of insulin decreased preference for the flavour of a paired glucose-containing solution versus the flavour associated with intact insulin signalling. Blocking insulin in the NAc shell also decreased consumption of a paired solution during one-bottle conditioning, whereas mock or control microinjections had no effect on consumption. These data suggest that insulin in the NAc shell plays a role in food preference. Previous studies have shown that intact DA signalling in the NAc is necessary for acquisition of flavour conditioning^37^,^38^, confirming a role for NAc DA in mediating the reinforcing effects of nutritive solutions. In this light, preference for the glucose solution paired with intact insulin availability argues against a primary effect of insulin on DAT-mediated DA uptake in the NAc shell, as this would be expected to decrease [DA]_o_ and therefore decrease the consumption of the control-paired flavour. Our results are also consistent with those from a previous study in which microinjection of insulin in the NAc shell increased the time the animals were engaged in oral sucrose self-administration, with a borderline increase in sucrose consumption^26^, which was opposite from the expected consequence of increased DA uptake. Overall, these behavioural data are consistent with the predicted influence of insulin on striatal ChIs and enhanced DA release. However, these results do not exclude the involvement of other elements of striatal microcircuitry in the monitored behaviours, given widespread InsR expression throughout striatum^1^,^14^.

The studies reported here provide the first evidence that insulin plays a role in communicating the caloric value, and therefore the rewarding effects of a meal, which has important implications for the influence of insulin in both underweight and obese subjects. Several studies indicate that the post-ingestive effects of food, regardless of whether taste transduction pathways are intact^47^, increase NAc DA release and positive reinforcement of behaviour^37^,^47^. Thus, the post-absorptive insulin response may encode the glycaemic yield of a meal and contribute to the reinforcement of food preferences and behaviours that enable consumption. However, extreme changes in circulating insulin levels and central InsR sensitivity could have a role in abnormal, as well as adaptive behaviour. For example, hypoinsulinaemia and compensatory upregulation of InsR sensitivity in FR subjects could be a factor in their disposition to binge^48^. Conversely, central insulin insensitivity in type II diabetes or obesity, mirrored here in OB rats, could contribute to a diminished sense of reward following ingestion, driving intake of foods with a high glycaemic index as compensation^49^,^50^. Therefore, either an increase or a decrease in striatal insulin sensitivity could contribute to pathological eating, resulting in binge eating and/or obesity.

Overall, our findings reveal a new role for insulin as a reward signal. Such a role contrasts with its known function as a satiety signal, including recent findings that insulin microinjected into the VTA can decrease hedonic feeding and preference for cues associated with food reward^23^,^24^. This raises the question of how seemingly opposing roles for insulin in these DA-dependent functions can be reconciled. The answer may be that these effects are complementary, rather than contradictory. The present results indicate that insulin in the striatum communicates the reward value of an ingested meal. A dual role in signalling satiety may simply allow insulin to serve the important purpose of ending a meal, while simultaneously establishing a memory of its nutritional and thus rewarding qualities, thereby reinforcing repetition of the ingestive behaviour.

## Methods

### Animal handling

Animal procedures were in accordance with the NIH guidelines and approved by the NYU School of Medicine Animal Care and Use Committee. All animals were on 12 h light:dark cycle, with lights on from 06:00 to 18:00; *ex vivo* slices were prepared between 08:00 and 12:00. Mechanistic studies in AL rats and mice were conducted in *ex vivo* slices from animals housed in pairs, whereas rats were singly housed for all diet group comparisons and for behavioural studies.

### Rat diet regimens

Adult male Sprague–Dawley rats (Taconic) were 8–10 weeks old at initiation of diet regimens lasting 21–30 days. Rats were semi-randomly assigned to the diet groups: subjects were ranked by initial weight, then each successive trio of rats was distributed randomly among the diet groups. AL rats had free access to rat chow for the same period as paired rats on FR or OB diets. All rats had free access to water. Food restriction was implemented as previously^51^; briefly, rats received 40–50% of AL intake of standard rat chow daily until body weight was reduced by 20%, after which food was titrated to maintain this weight. OB rats had free access to rat chow and chocolate Ensure, a highly palatable liquid with moderately high fat and sugar^52^.

### Forebrain *ChAT* knockout mice

Mice with a conditional floxed allele of *ChAT* (*ChAT*^flox^) were crossed with a *Nkx2.1*^Cre^ transgenic line to produce mice in which ablation of ACh synthesis is restricted to forebrain^32^. Non-mutant transgenic littermates were controls; their genotypes Cre^+^;*ChAT*^flox/+^ and Cre^−^;*ChAT*^flox/flox^ are referred to as ‘heterozygotes’. Adult male mice used for slice studies had *ad libitum* access to chow and water.

### *Ex vivo* slice preparation and physiological solutions

Rats or mice were deeply anaesthetized with 50 mg kg^−1^ pentobarbital (intraperitoneal (i.p.)) and decapitated. For voltammetry, coronal forebrain slices (300–400-μm thickness) were cut on a Leica VT1200S vibrating blade microtome (Leica Microsystems; Bannockburn, IL) in ice-cold HEPES-buffered artificial CSF (aCSF) containing (in mM): NaCl (120); NaHCO_3_ (20); glucose (10); HEPES acid (6.7); KCl (5); HEPES sodium salt (3.3); CaCl_2_ (2); and MgSO_4_ (2), equilibrated with 95% O_2_/5% CO_2_. Slices were then maintained in this solution at room temperature for 1 h before experimentation^30^,^32^,^53^. For electrophysiology, after anaesthetization, rats were perfused transcardially with ice-cold solution containing (in mM): sucrose (225); KCl (2.5); CaCl_2_ (0.5); MgCl_2_ (7); NaHCO_3_ (28); NaH_2_PO_4_ (1.25); glucose (7); ascorbate (1); and pyruvate (3), and equilibrated with 95% O_2_/5% CO_2_. Slices were cut in this solution, then transferred to a recovery chamber in modified aCSF containing (in mM): NaCl (125); KCl (2.5); NaH_2_PO_4_ (1.25); NaHCO_3_ (25); MgCl_2_(1); CaCl_2_ (2); glucose (25); ascorbate (1); pyruvate (3); and *myo*-inositol (4), equilibrated with 95% O_2_/5% CO_2_; this solution was initially at 34 °C, then allowed to cool gradually to room temperature^54^. All voltammetry and physiology experiments were conducted in a submersion recording chamber at 32 °C that was superfused at 1.5 ml min^−1^ with aCSF containing (in mM): NaCl (124); KCl (3.7); NaHCO_3_ (26); CaCl_2_ (2.4); MgSO_4_ (1.3); KH_2_PO_4_ (1.3); and glucose (10), and bovine serum albumin (BSA, 0.05–0.1 mg ml^−1^) equilibrated with 95% O_2_/5% CO_2_; slices were allowed to equilibrate in this environment for 30 min before experimentation.

### Fast-scan cyclic voltammetry

Evoked DA release studies were conducted using FCV in brain slices^32^,^53^ prepared from male rats or *ChAT* forebrain knockout mice and heterozygote controls (5–8 weeks). Studies in *ChAT* knockout mice were blinded, but rat diet groups had obvious phenotypes that precluded blinding. Voltammetric measurements were made with a Millar Voltammeter (available by special request to Dr Julian Miller at St Bartholomew’s and the Royal London School of Medicine and Dentistry, University of London). A conventional triangle waveform was used for FCV, with a scan range of −0.7 to +1.3 V (versus Ag/AgCl), scan rate of 800 V s^−1^, and sampling interval of 100 ms^30^,^32^,^53^. Data were acquired using a DigiData 1200B A/D board controlled by Clampex 7.0 software (Molecular Devices). DA release was evoked using a concentric stimulating electrode; stimulus pulse amplitude was 0.4–0.6 mA and duration was 100 μs^30^,^32^,^53^. Local single-pulse stimulation was used in NAc core and CPu; however, a brief high-frequency pulse train (five pulses at 100 Hz) was used to amplify evoked [DA]_o_ in NAc shell. Both stimulus paradigms evoke DA release that is action potential and Ca^2+^ dependent, unaffected by concurrently released glutamate and GABA^42^,^55^, and facilitated by concurrently released ACh^29^,^30^,^31^,^32^,^33^,^34^. To quantify evoked [DA]_o_, electrodes were calibrated with known concentrations of DA at 32 °C after each experiment in aCSF and in the presence of each drug used during a given experiment^53^.

Voltammetry experiments to assess the effect of insulin on evoked [DA]_o_ were obtained using either of two protocols. Initial experiments to determine the time course for the effect of insulin (Sigma, I5523) were performed by monitoring evoked [DA]_o_ every 5 min in a single site. Insulin was applied after consistent evoked [DA]_o_ was obtained (typically 4–5 measurements); the effect of insulin was maximal after 50–60 min, and then evoked [DA]_o_ remained at this level for the duration of the experiment (typically 90 min total insulin exposure; Fig. 1c). Subsequently, the effect of insulin was assessed by recording evoked [DA]_o_ at 4–5 discrete sites in slices (+1.5 mm from bregma) in each of three striatal subregions under control conditions (aCSF or aCSF plus drug) and again at the time of maximal insulin effect (sampling over 60–80 min), then these samples were averaged for each subregion. The effect of insulin diminished with time after slice preparation; to minimize time *ex vivo* and to optimize animal use, typically, two slices from a given animal were tested in the recording chamber at the same time. Drugs used to challenge the effect of insulin were applied 15 min before insulin via superfusing aCSF, including HNMPA trisacetoxymethyl ester (HNMPA-AM_3;_ Enzo Life Sciences), S961 (Novo Nordisk), LY294002 (Sigma), picropodophyllotoxin (PPP; Tocris), mecamylamine (Tocris) and DHβE (Tocris). As described in Results, possible altered sensitivity of nicotinic ACh receptors among diet groups was tested by comparing the ratio of peak [DA]_o_ evoked by 5 p (100 Hz) with that evoked by 1 p evoked (5 p:1 p ratio)^30^,^31^ in NAc core in the presence of 0–500 nM nicotine (Sigma).

### Determination of *V*_max_ from evoked [DA]_o_ transients in striatal slices

To evaluate insulin-induced changes in DAT-mediated DA uptake, the initial portion of the falling phase of evoked [DA]_o_ curves was fitted to the Michaelis–Menten equation to extract *V*_max_ (maximal uptake rate constant)^56^. *K*_m_ (which is inversely related to the affinity of the DAT for DA) was fixed at 0.2 μM and is known to be similar across striatal subregions^57^ and unaffected by insulin (see Supplementary Fig. 4 caption).

### Whole-cell recording

Brain slices were prepared from 29- to 35-day-old male rats; recording conditions were identical to those used in DA release studies. Whole-cell current-clamp recordings used conventional methods^54^. Striatal ChIs were visualized using an Olympus BX51WI microscope (Olympus America, Center Valley, PA) with infrared differential-interference contrast optics and a × 40 water-immersion objective. The pipette solution contained (in mM): K-gluconate (129); KCl (11); HEPES (10); MgCl_2_ (2); EGTA (1); Na_2_-ATP (2); Na_3_-GTP (0.3); and adjusted to pH 7.2–7.3 with KOH. For recorded neurons to be assessed for ChAT immunoreactivity, 0.3% biocytin was included in the pipette solution and the neurons recorded briefly (∼5 min) to minimize dilution of intracellular contents. Pipette resistance was ∼3–5 MΩ. Recordings were obtained using an Axopatch 200B amplifier (Molecular Devices, Sunnyvale CA) and low-pass filtered at 2 kHz. ChIs were identified by established electrophysiological criteria^28^; most were tonically active initially, but activity diminished variably after patching. However, responsiveness to current injection was generally robust and consistent over time, and was therefore used to investigate the effect of insulin on ChI excitability (see Results). In experiments to examine the role of InsRs and IGF-1Rs in this response, either HNMPA or PPP was applied for at least 20 min before a ChI was patched. Maximal effects of insulin alone were typically observed after ∼16 min of exposure, although in some cells, the increase was not maximal until 50 min or longer. Moreover, in four of six neurons recorded in PPP, insulin caused an initial decrease in spike number before recovering to and exceeding starting spike number. Consequently, in all experiments, the effect of insulin was quantified by comparing the maximum effect on spike number with the number of spikes evoked immediately before insulin application. The apparent difference in the time to reach peak effect could reflect a number of factors, including the depth of the recorded cell in the slice. Evoked action potentials were also recorded in ChIs in the absence of insulin at comparable time points.

### High-performance liquid chromatography

The DA content of rat striatal slices (400-μm thickness) was determined using high-performance liquid chromatography with electrochemical detection^58^. Slice pairs were equilibrated for 30 min at 32 °C in aCSF, and then one slice per pair was incubated for an additional 60 min at 32 °C in aCSF while the other was incubated in aCSF with 10 or 30 nM insulin. For diet group comparisons, striatal tissue was collected between 30–60 min post-recovery. Following incubation, excess aCSF was carefully removed from slices, a sample of striatal tissue (7–10 mg) was weighed, frozen on dry ice and then stored at then stored at −80 °C. On the day of analysis, samples were sonicated in ice-cold, eluent, deoxygenated with argon^58^, spun in a microcentrifuge for 2 min, and the supernatant injected directly onto the HPLC column (BAS, West Lafayette, IN); the detector was a glassy carbon electrode set at 0.7 V versus Ag/AgCl.

### Immunohistochemistry

For immunohistochemical labelling, rats were anaesthetized with sodium pentobarbital (50 mg kg^−1^, i.p.), then perfused transcardially with PBS (154 mM NaCl in 10 mM phosphate buffer, pH 7.2) followed by 4% paraformaldehyde in this PBS; brains were removed and coronal sections (20 μm) were cut and processed conventionally^27^,^59^. Immunofluorescence images were obtained with a Nikon PM 800 confocal microscope equipped with a digital camera controlled by Spot software (Diagnostic Instruments Inc.) and using a × 100 objective (numerical aperture=1.4) or with a Zeiss LSM 510 confocal microscope using a × 63 objective (numerical aperture=1.2). The lasers were Argon (488 nm), He/Ne (543 nm) and He/Ne (633 nm). Appropriate filters for each laser were selected by the confocal microscope software. Pinhole size varied with the objective used and section thickness selected in *z*-stack generation; we chose the optimum pinhole value indicated by the software (typically 30 μm). Digital files were analysed with deconvolution software (AutoQuant Imaging), with final images processed using Adobe Photoshop 7.0. All images were adjusted for brightness and contrast; such adjustments were made uniformly to all parts of the image. Striatal DA axons were identified using two TH antibodies: polyclonal AB152 rabbit anti-TH (1:800) and monoclonal MAB318 mouse anti-TH (1:500) (both from Chemicon). Three InsR antibodies were used: sc-57342 and sc-09 (1:100; Santa Cruz), and PP5 (a gift from Pfizer). The specificity of each has been demonstrated previously^60^,^61^, and was confirmed in the present studies by the absence of immunolabelling with antibodies sc-57342 or PP5 in the presence of the corresponding blocking peptide. ChAT antibody was AB144 (1:200; Millipore), and biotin was from Vector (1:200). Secondary antibodies used were donkey anti-rabbit Alexa 488 (Invitrogen), or donkey anti-rabbit Cy2 (Jackson Laboratory, Bar Harbor, ME), donkey anti-goat Cy3 (Jackson) and donkey anti-mouse Cy5 (Jackson).

To evaluate co-localization of InsRs in TH+ axons, we used methods described previously to identify the presence of Kir6.2, the pore-forming subunit of ATP-sensitive K^+^ channels in DA axons^27^. Puncta representing InsRs were distributed throughout adjusted images, indicating that superimposition with TH immunoreactivity may occur to some degree by chance. To test this assumption, we counted InsR/TH superimpositions in 42 independent fields in three NAc immunolabelled sections from two rats. The InsR digital files were then rotated 90° clockwise and the counts repeated; rotation decreased the number of InsR puncta that co-localized with TH in most fields (see Results). The decrease in the number of superimpositions with rotation^27^ indicated the proportion of InsR puncta in each striatal field associated with DA axons.

### Blood glucose and insulin ELISA

Trunk blood was collected at time of decapitation for slice studies. Blood glucose was determined immediately with a standard blood glucose monitor. For insulin, additional blood was collected in EDTA-containing tubes and centrifuged at 1,500*g* for 15 min; supernatant (plasma) was collected and stored at −80 °C until processing with an ALPCO Rat Insulin ELISA kit.

### Cannula placement and histological verification

Forty one adult male Sprague–Dawley rats (Taconic and Charles River) initially weighing 350–425 g were anaesthetized with ketamine (100 mg kg^−1^, i.p.) and xylazine (10 mg kg^−1^, i.p.) and stereotaxically implanted with two chronically indwelling guide cannulae (26 gauge) placed bilaterally 2.0 mm dorsal to infusion sites in the NAc medial shell^62^ (1.6 mm anterior to bregma; 2.1 mm lateral to the sagittal suture, tips angled 8° towards the midline, 5.8 mm ventral to skull surface). Rats were given banamine (2.0 mg kg^−1^, subcutaneous) as a post-surgical analgesic following recovery from anaesthesia and the morning after. One week after surgery, rats were placed on FR (described above) and maintained at 80% of their post-surgical recovery weight for the remainder of the study. Cannula placement was determined histologically after completion of behavioural testing. Each rat was killed with CO_2_, decapitated and the brain removed and fixed in 10% buffered formalin for >48 h. Frozen coronal sections (40-μm thickness) were cut on a Reichert-Jung Cryostat, thaw mounted on gelatin-coated glass slides, and stained with cresyl violet. Data from a given rat were used only if both cannulae were within the medial NAc shell^62^ (including the shell/core or shell/olfactory tubercle border) (Supplementary Fig. 5); based on these criteria, two rats were excluded from the final analysis.

### Flavour-preference conditioning pre-exposure

Rats received one overnight (in home cage) and six 30-min-per-day sessions of pre-exposure (in testing chambers) to 0.2% sodium saccharin (Sigma) in water with a 48-h interval between sessions. Rats then received two 5-min-per-day sessions of exposure to 0.2% sodium saccharin in 0.05% unsweetened grape or cherry Kool-Aid (Kraft Foods) in water. For the first Kool-Aid pre-exposure session, half of the rats received cherry-flavoured solution, and the other half received grape-flavoured solution. The flavours were reversed on the second Kool-Aid pre-exposure session to ensure that all rats sampled each flavour. Intake was measured for all pre-exposure sessions. Testing chambers were clear plastic cages with fresh bedding. For all pre-exposure sessions, rats had access to the same solution on both sides of the chamber. Except for the overnight pre-exposure session, all sessions were conducted in a behavioural procedure room, with a 30-min habituation period before any training or testing.

### One-bottle conditioning

Previous studies have shown that microinjection of InsAb into ventromedial hypothalamus can block the effect of insulin on feeding behaviour and glucagon secretion^63^,^64^. Here we used this approach to assess a possible role of insulin in the reinforcement of food choice. Rats were semi-randomly assigned based on average pre-exposure intake volume into two groups, control or experimental (InsAb). In the control group, rats received vehicle (microinjection PBS; 137 mM NaCl and 2.7 mM KCl in 10 mM phosphate buffer) or IgG (Abcam ab81032; 0.5 μg μl^−1^ in PBS, as received) microinjection in NAc shell before consuming one of the two flavoured solutions, and a mock microinjection before consuming the other flavoured solution. In the experimental group, rats received NAc shell microinjection of InsAb (Abcam ab46707; 0.5 μl of 1 μg μl^−1^ in PBS, as received) before exposure to one flavoured solution, and mock microinjection prior exposure to the other. Two sets of subjects with alternation between fluid microinjection and mock microinjection were used, so that the total number of microinjections was limited to four, thereby minimizing possible tissue damage and loss of sensitivity at the microinjection site^65^. For fluid microinjection, control solution or InsAb was loaded into two 30-cm lengths of PE-50 tubing attached at one end to 5 μl Hamilton syringes filled with distilled water and at the other end to 31-gauge injector cannulae, which extended 2.0 mm beyond the implanted guides. The 0.5 μl infusion volumes were delivered over 90 s at a rate of 0.005 μl s^−1^; the injector was left in place for ∼60 s to allow time for diffusion, then the injector was replaced with the stylet.

Rats were transferred directly to the behavioural chambers within 2 min of completion of the microinjection or mock microinjection. Conditioning solutions contained 0.2% sodium saccharin, 0.05% unsweetened grape or cherry Kool-Aid, and 0.8% glucose. Solution access was limited to 30 min per session. Paired flavour and side of the chamber with drinking access were semi-randomly assigned and counterbalanced in each group. The interval between microinjections was at least 72 h, alternating between infusion and mock sessions for a total of eight conditioning sessions.

### Two-bottle preference test

Forty-eight hours after the last conditioning session, rats were placed in testing chambers with simultaneous access to both conditioning flavours; solutions were 0.2% sodium saccharin in 0.05% grape or cherry Kool-Aid, without glucose. Testing occurred over 2 days (60 min per day). The position of the drinking tube containing mock-paired or infusion-paired solution was alternated to ensure that each rat was tested for consumption of each solution on both sides of the cage. Intake of each flavoured solution was averaged for the two test days to determine preference.

### [^3^H]DA uptake in striatal synaptosomes to assess InsAb efficacy

Striatal synaptosomes^21^,^66^ were prepared from AL rats (male, 350–400 g), with NAc (shell and core) and CPu dissected and prepared separately. Tissue from each region was homogenized in 15 volumes of an ice-cold 0.32 M sucrose solution in a glass homogenizer with motor-driven Teflon pestle; after rinsing and centrifugation, the final pellet was re-suspended in ice-cold 0.32 M sucrose^21^,^66^. Before initiating the [^3^H]DA uptake assay^66^, synaptosomal aliquots in a total volume of 180 μl of uptake buffer were incubated in a shaker for 15 min at 30 °C in the presence or absence of 30 nM insulin, in vehicle (PBS) or in InsAb (final dilution 1:500), in IgG (final dilution 1:500) or in vehicle. Uptake buffer contained (in mM): NaCl (122); Na_2_HPO_4_ (3); NaH_2_PO_4_ (15); KCl (5); MgSO_4_ (1.2); glucose (10), CaCl_2_ (1); nialamide (0.01); tropolone (0.1); and ascorbic acid (0.001), pH 7.4. Uptake of [^3^H]DA was initiated by rapidly dispensing 20 μl of each synaptosomal suspension into the 96-well plates with varying concentrations of DA (0.003–1.0 μM) and [^3^H]DA (5 nM); after 5 min in a plate-shaker at 25 °C, uptake was terminated by cold, rapid vacuum filtration^66^. Counts per well were converted to pmoles, then corrected to mg of total protein per minute. All assays were performed in triplicate and repeated at least four times; *V*_max_ and *K*_m_ were calculated using Biosoft Kell Radlig software (Cambridge, UK).

### Statistical analysis

Data are given as means±s.e.m.; significance was assessed using paired or unpaired Student’s *t*-tests or ANOVA, unless indicated otherwise. For voltammetry data, *n* is number of recording sites, given that site-to-site variability within a striatal subregion is greater than inter-animal or inter-slice variability^30^,^32^,^55^,^56^; animal number is noted for each data set. The EC_50_ for the effect of insulin and nicotine on peak-evoked [DA]_o_ was calculated using Prism 6.0 (GraphPad Software Inc., La Jolla, CA). For electrophysiological data, statistical significance was assessed using paired *t*-tests or a Wilcoxon test in Prism 6.0, or a mixed two-way ANOVA in SAS 9.3 (SAS Institute Inc., Cary, NC). For evaluation of insulin involvement in flavour-preference conditioning, two full studies were completed using protocols that were identical with the exception of the vehicle treatment used. In the first study, 10 rats received vehicle infusions of PBS and 9 received infusions of InsAB. In the second study, 10 rats received vehicle infusions of IgG and 10 received infusions of InsAb. There was no significant difference between the two vehicle groups (PBS or IgG) during infusion-conditioning sessions (*F*_19_=0.619, mixed two-way ANOVA) with repeated measures on infusion-conditioning session) or at test (*F*_19_=0.012, two-way mixed ANOVA with repeated measures on flavour). Consequently, the two experiments were combined for analysis. For this analysis, a 2 × 4 mixed ANOVA (with repeated measures on infusion-conditioning day) was used to determine the effects of microinjection treatment during conditioning, followed by protected *t*-tests (one tailed to determine in which conditioning sessions microinjection treatment decreased intake volume). The same analysis was completed for mock conditioning sessions. To determine the effect of conditioning treatment during a two-bottle flavour-preference test, data were analysed using a mixed two-way ANOVA (with repeated measures on flavour), followed by protected *t*-tests (one tailed to test the hypothesis that InsAb would decrease preference).

## Additional information

**How to cite this article:** Stouffer, M. A. *et al*. Insulin enhances striatal dopamine release by activating cholinergic interneurons and thereby signals reward. *Nat. Commun*. 6:8543 doi: 10.1038/ncomms9543 (2015).

## Supplementary Material

Supplementary Information Supplementary Figures 1-5 and Supplementary Reference

## Figures and Tables

**Table 1:** Insulin at physiological concentrations (30 nM) increases *V*_max_ for DAT-mediated uptake in striatal slices.

*V*_max_ (μM s^−1^)						
	Control	*n*	Insulin (30 nM)	*n*	Insulin (100 nM)	*n*
NAc shell	0.88±0.04	25	1.09±0.09*	21	0.82±0.05	23
	*R*^2^=0.9125		*R*^2^=0.9392		*R*^2^=0.9014	
NAc core	2.06±0.07	73	2.32±0.08*	61	2.20±0.08	36
	*R*^2^=0.9937		*R*^2^=0.9929		*R*^2^=0.9912	
CPu	3.23±0.08	62	3.63±0.15*	40	3.39±0.09	29
	*R*^2^=0.9902		*R*^2^=0.9924		*R*^2^=0.9946	

*V*_max_ values for DAT-mediated uptake after evoked striatal DA release were determined from evoked [DA]_o_ records using a fixed *K*_m_ of 0.2 μM for all regions and conditions. Data are means±s.e.m, *n*, number of sites, *R*^2^ indicates goodness of fit (*P<0.05 versus control, one-way ANOVA followed by Dunnett’s *post hoc* test).

**Table 2:** Final body weight, weight change, plasma insulin and blood glucose values of rats on AL, OB or FR diet.

Diet group	Final body weight (g)	Per cent weight gain or loss	Plasma insulin (ng ml^−1^)	Blood glucose (mg dl^−1^)
AL	451±5	23.6±1.8	1.11±0.11	125±4
OB	523±10***	36.2±1.9***	1.51±0.18	125±6
FR	304±3***	−21.9±0.3***	0.44±0.08***	117±5

Data are means±s.e.m. (*n*=12–24 rats, ***P<0.001 versus AL, one-way ANOVA followed by Tukey HSD).
