# Supplementary material for: Insulin enhances striatal dopamine release by activating cholinergic interneurons and thereby signals reward
Source: Nat Commun. 2015 Oct 27;6:8543. doi: 10.1038/ncomms9543 (PMC4624275; doi:10.1038/ncomms9543)
Supplement: Supplementary Information — Supplementary Figures 1-5 and Supplementary Reference [file ncomms9543-s1.pdf]

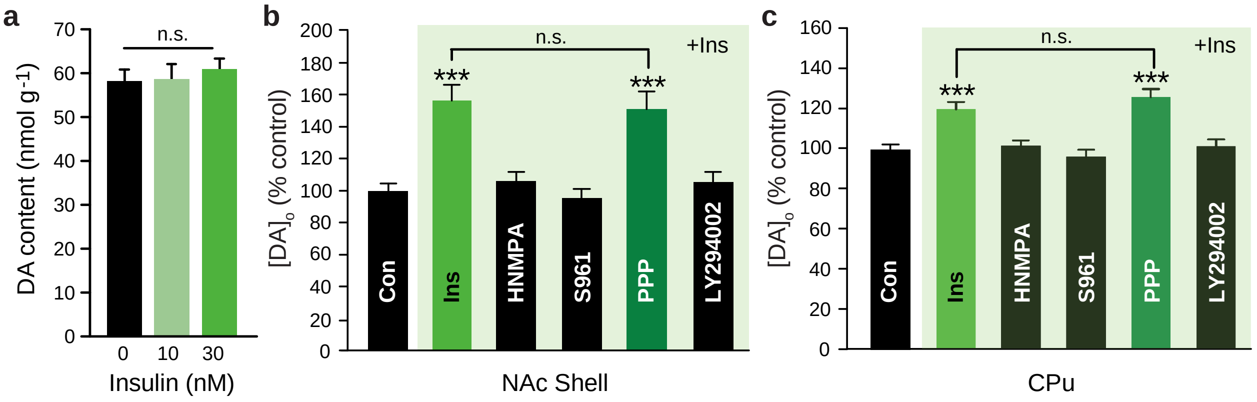

Supplementary Figure 1. Insulin increases DA release without affecting DA content via InsRs. a) Tissue DA content analyzed by HPLC from striatal slices incubated in the absence or presence of 10 or 30 nM insulin for 60 min. There was no significant difference in tissue DA content between any treatment (*n* = 17-19 samples from 3-5 rats per condition, $F_{2,55} = 0.263$, $P > 0.7$, one-way ANOVA). **b-c**) InsR inhibitor HNMPA (5 μM), InsR antagonist S961 (1 μM), and PI3K inhibitor LY294002 (1 μM) each completely prevented the effect of insulin (30 nM) on increasing evoked $[DA]_o$. In the presence of the IGF-1R inhibitor PPP (1 μM), there was a significant increase in evoked $[DA]_o$ (***$P < 0.001$) that was not significantly different from that of insulin alone ($P > 0.9$; n =21-28 (NAc shell) and n = 24-62 (CPu) sites per subregion per drug or insulin concentration from 3-6 rats; one-way ANOVA, Tukey HSD).

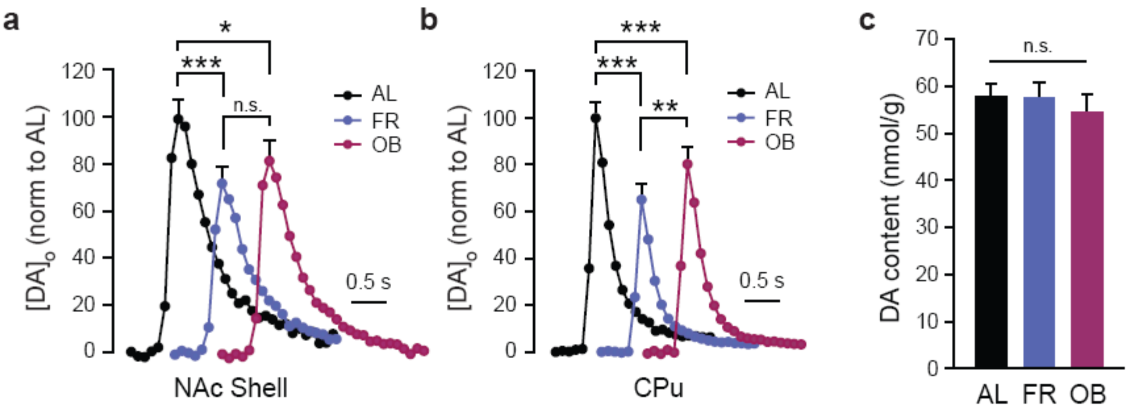

Supplementary Figure 2. Evoked $[DA]_o$ in NAc shell and CPu is decreased by chronic food restriction (FR) or an obesogenic (OB) diet. a-b) Average single-pulse evoked $[DA]_o$ in NAc shell and CPu under control conditions in FR and OB rats normalized to 100% peak AL (*ad-libitum* control diet). Data are means ± s.e.m. **a**) Evoked $[DA]_o$ in NAc shell differed significantly among diet groups (n = 40-42 sites from 5-6 rats per diet group, $F_{2,156}$ = 9.723, one-way ANOVA followed by Tukey HSD) with a decrease of 26 ± 4% in FR and 15 ± 5% in OB *vs.* AL rats. Evoked $[DA]_o$ did not differ between OB *vs.* FR (P < 0.18). **b**) Evoked $[DA]_o$ in CPu differed significantly among diet groups (n = 40-48 sites from 5-6 rats per diet group, $F_{2,122}$ = 28.181, one-way ANOVA followed by Tukey's multiple comparisons) with a decrease of 35 ± 6% in FR and 19 ± 8% in OB *vs.*AL rats. Evoked $[DA]_o$ was also significantly lower in FR *vs.*OB (*P < 0.001;**P < 0.01; ***P < 0.001). **c**) Tissue DA content analyzed by HPLC from striatal slices from AL, FR, and OB rats. There was no difference in tissue DA content among diet groups (n = 13-18 samples from 3 rats per diet group, $F_{2,45}$ = 0.325, P > 0.7, one-way ANOVA).

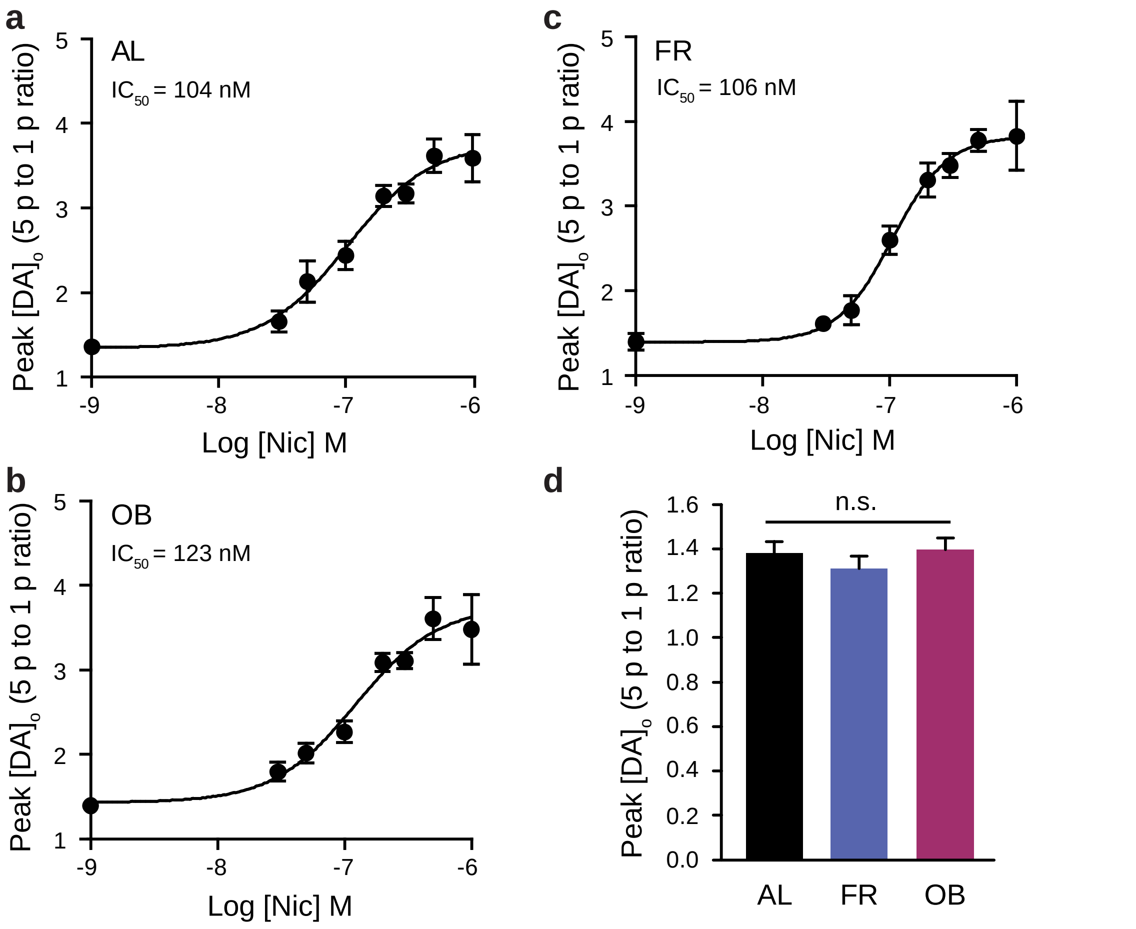

Supplementary Figure 3. nAChR sensitivity is unaltered by chronic food restriction (FR) or an obesogenic (OB) diet vs. *ad libitum* (AL) feeding. a-d) The ratio of peak 5 p (100 Hz) to 1 p evoked $[DA]_o$ (5 p:1 p ratio) in NAc core in the presence of 0-500 nM nicotine in AL (**a**), FR (**b**), and OB (**c**)**.** EC_{50} values for the effect of nicotine did not differ among diet groups (n = 6-12 same-site stimulus pairs from 3-5 rats per condition, $F_{2,253} = 1.243$; $P = 0.29$, one-way ANOVA). **d**) Under control conditions, 5 p:1 p ratios also did not differ among diet groups (n = 22-24 same-site stimulus pairs from 3-5 rats per condition, $F_{2,67} = 0.691$; $P = 0.51$, one-way ANOVA).

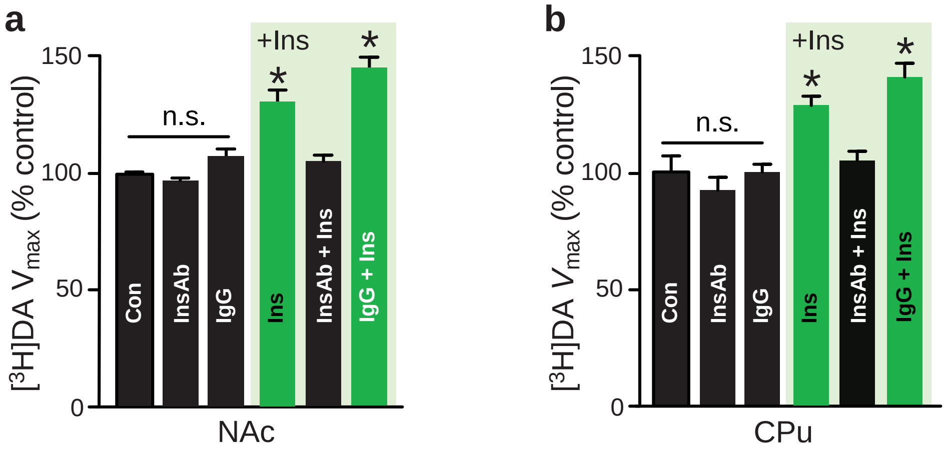

Supplementary Figure 4. Insulin-induced increases in DAT-mediated DA uptake in synaptosomes from AL rat NAc or CPu is prevented by InsAb but not control IgG. Synaptosomes prepared from **a**) NAc (shell and core) or **b)** CPu were incubated in for 15 min at 30ºC in the absence or presence of insulin (Ins; 30 nM) under control conditions (Con; including antibody vehicle, PBS), and in the presence of a non-specific antibody IgG (1:500 dilution) or InsAb (1:500 dilution). Synaptosome aliquots from each group were then tested in a [^{3}H]DA uptake assay to determine V_{max} for DAT-mediated uptake under each condition. Data are means ± s.e.m. of at least 4 experiments, each performed in triplicate. For these comparisons, data were normalized, with control V_{max} taken as 100% for each striatal subregion; absolute V_{max} for CPu was ~5-fold higher than that for NAc. Consistent with the increase seen in V_{max} for DA uptake in striatal slices after insulin (30 nM) exposure (**Table 1**), a significant insulin-induced increase in V_{max} *vs*. control was seen in synaptosomes from NAc (**a**) and CPu (**b**) (*$P < 0.05$ *vs.* control, one-way ANOVA followed by Dunnett's post-hoc test). An insulin-induced increase in V_{max} *vs.* control was also seen in the presence of IgG (*$P < 0.05$) in both regions. However, the effect of insulin was lost in the presence of InsAb ($P > 0.05$). The presence of IgG or InsAb alone did not alter V_{max} ($P > 0.05$ *vs.* control). Further kinetic analysis revealed that K_m was unaltered by any of these conditions ($P > 0.05$ for all comparisons, one-way ANOVA).

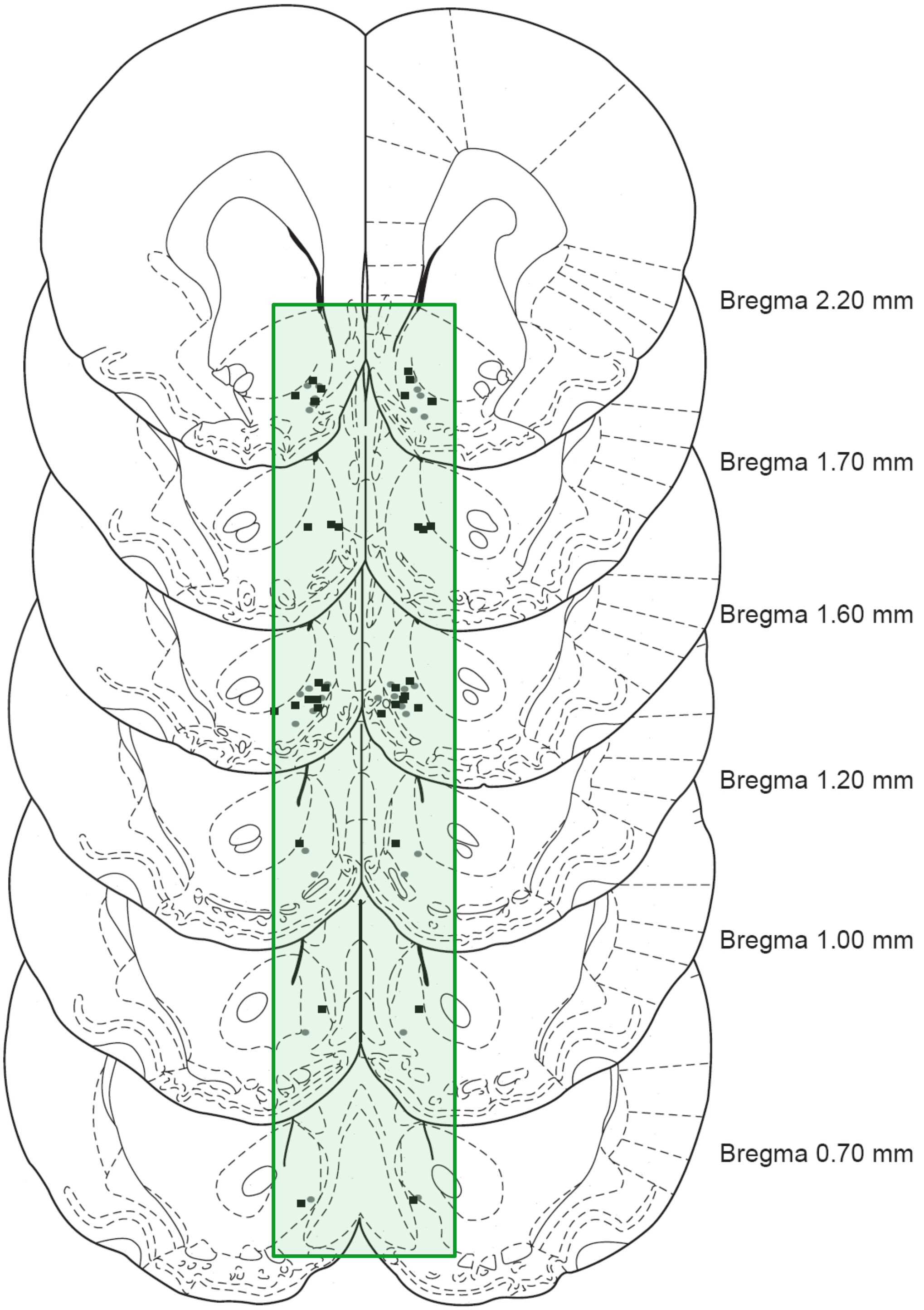

Supplementary Figure 5. Location of bilateral cannula placement in the NAc shell for flavor preference subjects. Placement is indicated for rats included in data analysis from the InsAb group (■) and control group (●); rats (*n* = 2) with cannula placement outside of the NAc shell excluded from the analysis and from this figure. Coronal sections were modified from ref. 1.

Reference

1. Paxinos, G. & Watson, C. *The Rat Brain in Stereotaxic Coordinates*, *Sixth Edition* (Academic Press, New York, NY, 2007).
